# Folic-acid metabolism and DNA-repair phenotypes differ between neuroendocrine lung tumors and associate with aggressive subtypes, therapy resistance and outcome

**DOI:** 10.18632/oncotarget.7737

**Published:** 2016-02-26

**Authors:** Robert Fred Henry Walter, Fabian Dominik Mairinger, Robert Werner, Claudia Vollbrecht, Thomas Hager, Kurt Werner Schmid, Jeremias Wohlschlaeger, Daniel Christian Christoph

**Affiliations:** ^1^ Ruhrlandklinik Essen, West German Lung Centre, University Hospital Essen, University of Duisburg-Essen, Essen, Germany; ^2^ Institute of Pathology, University Hospital Essen, University of Duisburg-Essen, Essen, Germany; ^3^ Institute of Pathology, Division of Molecular Pathology, Charité, Berlin, Essen, Germany; ^4^ Department of Pathology, Helios Klinikum Emil von Behring, Berlin, Germany; ^5^ Institute of Pathology, Ev.-Luth. Diakonissenkrankenhaus Flensburg, Flensburg, Germany; ^6^ Department of Medical Oncology, West German Cancer Centre, University Hospital Essen, University of Duisburg-Essen, Essen, Germany

**Keywords:** lung cancer, neuroendocrine pulmonary tumors, folic acid metabolism, DNA repair, NanoString nCounter

## Abstract

**Purpose:**

25% of all lung cancer cases are neuroendocrine (NELC) including typical (TC) and atypical carcinoid (AC), large-cell neuroendocrine (LCNEC) and small cell lung cancer (SCLC). Prognostic and predictive biomarkers are lacking.

**Experimental Design:**

Sixty patients were used for nCounter mRNA expression analysis of the folic-acid metabolism (*ATIC*, *DHFR*, *FOLR1*, *FPGS*, *GART*, *GGT1*, *SLC19A1*, *TYMS*) and DNA-repair (*ERCC1*, *MLH1*, *MSH2*, *MSH6*, *XRCC1*). Phenotypic classification classified tumors (either below or above the median expression level) with respect to the folic acid metabolism or DNA repair.

**Results:**

Expression of *FOLR1*, *FPGS*, *MLH1* and *TYMS* (each p<0.0001) differed significantly between all four tumor types. *FOLR1* and *FPGS* associated with tumor differentiation (both p<0.0001), spread to regional lymph nodes (*FOLR1* p=0.0001 and FPGS p=0.0038), OS and PFS (*FOLR1* p<0.0050 for both and *FPGS* p<0.0004 for OS).

Phenotypic sorting revealed the Ft-phenotype to be the most prominent expression profile in carcinoids, whereas SCLC presented nearly univocal with the fT and LCNEC with fT or ft. These results were significant for tumor subtype (p<0.0001).

**Conclusions:**

The assessed biomarkers and phenotypes allow for risk stratification (OS, PFS), diagnostic classification and enhance the biological understanding of the different subtypes of neuroendocrine tumors revealing potential new therapy options and clarifying known resistance mechanisms.

## INTRODUCTION

Lung cancer is still the leading cause of cancer deaths worldwide with a poor five-year survival of approximately 15% [1]. Twenty-five percent of all lung tumors belong to the group of neuroendocrine tumors [2] including typical carcinoid (TC) and atypical carcinoid (AC), large-cell neuroendocrine cancer (LCNEC), and small-cell lung cancer (SCLC). Pattern of metastasis and survival rates differ significantly between these subtypes [3, 4].

According to the World Health Organization (WHO) classification from 2004, LCNEC is considered as a type of non-SCLC [5]. However, it shares clinical features with SCLC such as a five-year survival of 15% up to 57% depending on the reporting source [2, 3]. Even when diagnosed at an early stage, LCNEC and SCLC show the poorest clinical outcome compared to other malignant lung tumors due to the aggressiveness of these cancer types, but the molecular characteristics of these tumors still remain largely unknown [6]. They occur almost exclusively in patients with a history of smoking and grow very rapidly, whereas lung carcinoids occur frequently in never smokers and younger patients [4, 7-9].

A platinum-based combination chemotherapy is currently the standard first-line therapy for patients with advanced Epidermal Growth Factor Receptor (*EGFR*) gene and Anaplastic Lymphoma Kinase (*ALK*) gene wild-type non-small cell lung cancer (NSCLC) [10]. The multitarget antifolate pemetrexed is used in combination with cisplatin [11] or carboplatin [12] in non-squamous NSCLC (including metastatic atypical carcinoids and LCNEC) [13] and is often also administered after platinum-based chemotherapy as continuous maintenance therapy, as single agent after progression of first-line therapy [14, 15] or given as first-line therapy to patients who are medically unfit for platinum-based combination chemotherapy [16]. Three transporters are identified for the transport of antifolates into eukaryotic cells: the solute carrier family 19, member 1 (SLC19A1, also known as reduced folate carrier), the solute carrier family 46, member 1 (SLC46A1, also known as proto-coupled folate transporter), and the folate receptors of which folate receptor 1 (FOLR1) being the most widely studied isoform [17]. Pemetrexed inhibits folate-dependent enzymes such as thymidylate synthetase (TYMS), dihydrofolate reductase (DHFR) and glycinamide ribonucleotide formyltransferases (GART) [18]. Once pemetrexed is taken up by cells, it undergoes ATP-dependent polyglutamylation catalysed by folylpolyglutamate synthase (FPGS), regulated by the reverse mechanism through gamma-glutamyltransferase 1 (GGT1). Polyglutamylation results in more negatively charged molecules leading to higher intracellular concentration of pemetrexed [19]. Furthermore, pemetrexed shows a more than 60-fold higher activity when polyglutamylated than in its unmodified form [20]. Many of the enzymes mentioned above are involved in primary and acquired resistance mechanisms against pemetrexed [21].

Mechanism of platinum cytotoxicity includes the formation of bulky DNA adducts (generation of a chemically altered base in DNA by covalent binding of platinum [22, 23] leading to both inter- and intra-strand cross-link accumulation in DNA [23, 24]. Finally, platinum-compounds prevent normal cell replication and trigger apoptosis [25] unless adducts from genomic DNA are repaired [24].

Possible ways in which cancer cells develop a platinum-resistance include an efficient DNA-repair mechanism. The processing of cross-links in mammalian cells is not clearly understood. However, it is known that their processing may involve components belonging to different DNA repair pathways, including the nucleotide excision repair (NER) and the mismatch repair (MMR) pathway [26]. NER is capable of removing numerous types of DNA helix-distorting lesions induced by platinum [27]. The structure specific endonuclease excision repair cross-complementation group 1 (ERCC1) performs an essential late step in the NER process [27] and is the rate-limiting member of the NER pathway [23, 24]. Homologous to ERCC1 in NER, X-ray repair complementing 1 (XRCC1) is the key member of the base-excision repair (BER) pathway [28, 29]. In contrast, MMR is one of the major DNA-repair pathways, which is responsible for the repair of single-base or nucleotide mismatches and insertion-deletion loops.

MutS homologue 2 (MSH2) protein belongs to the MMR pathway and binds to platinum-induced DNA interstrand cross-links recognized by the MutSα complex, a MSH2/MSH6 heterodimer [30, 31], thereby initiating their excision and repair [26]. Additionally, during the recombinational repair processing of interstrand cross-links, MSH2 cooperates with several components of DNA damage repair pathways, including ERCC1 [26]. MutL homologue 1 (MLH1) protein is also a key component in the MMR pathway being involved in mismatch strand excision and subsequent repair. In malignant pleural mesothelioma, expression levels of enzymes involved in NER and MMR pathways were correlated to each other [32].

Defects in DNA-MMR have been shown to be a mechanism of resistance to cisplatin both *in vivo* and *in vitro* [23]. Furthermore, reduced expression levels of MLH1 or MSH2 at the protein and transcriptional level have been reported in some thoracic cancers including lung cancer [26, 33, 34], but for neuroendocrine lung tumors a lack of prognostic and predictive biomarkers associated with response or outcome following chemotherapy limits the improvement of current systemic therapies. A significant amount of these tumors shows a primary or acquired resistance to platinum or antifolate containing therapeutic regimes.

The main aims of our study were to find markers supporting the procedure of pathological diagnosis making in histologically difficult cases and distinguishing rare carcinoids (TC and AC) with metastatic spread, which need systemic treatment, from the less aggressive ones. Furthermore, we aimed at the analyses of prognostic and predictive markers for aggressive subtypes of NELC. Within this group of tumors (e.g. LCNEC) it is of clinical importance to find markers for the correct interpretation of the biological aggressiveness to adapt treatment approaches. Finally, we wanted to characterize the putative resistance mechanisms against platinum or antifolate containing therapeutic regimes using the NanoString nCounter system. The nCounter technology is a hybridization-based digital detection method that can be used to analyze mRNA, miRNA and copy number variations (CNV) [35-37]. Two sequence-specific probes were used to detect the target nucleic acid covering approximately 100 nt of the gene of interest. 100 nt were identified as reproducibly detectable/amplifiable length for FFPE-derived nucleic acids [38, 39].

## RESULTS

### Study population

**Figure 1:**
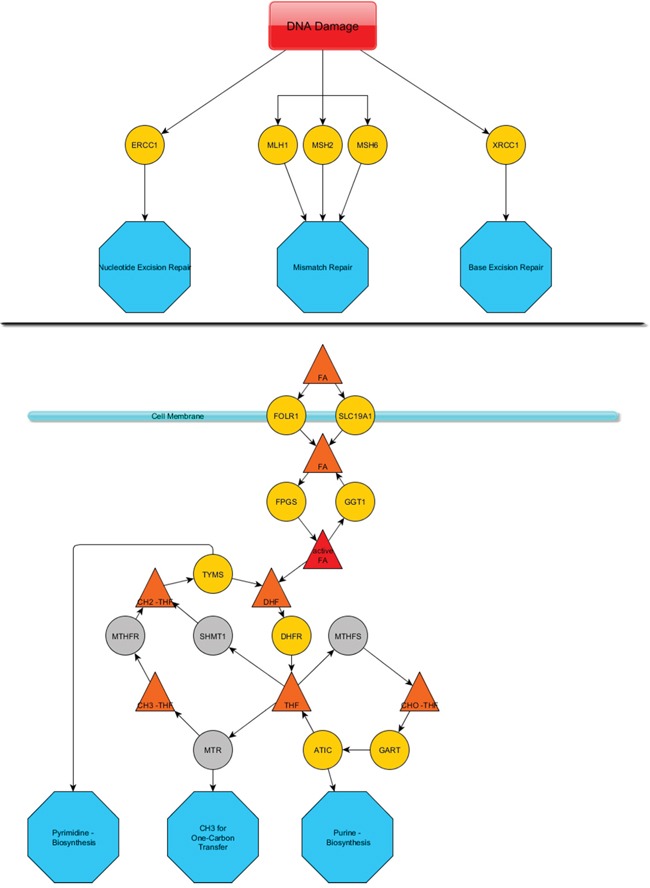
Sketch of the investigated DNA repair members and folic acid pathway The upper line **A.** depicts the DNA damage response members that were investigated and their mode of action (blue octahedrons). The lower line **B.** presents a summary of the folic acid metabolism pathway. Yellow spheres represent mRNAs that were assessed via the nCounter technology. Grey spheres were not assessed. A cell membrane is pictured by a pale blue line associated with two spheres, which represent transmembrane proteins. Triangles depict conjugates of folic acid that is substrate/product of the mentioned enzymes. Blue octahedrons represent the final deployment of the modified folic acids.

The mean age at date of diagnosis was 58.6 years (median age: 59 years; range 19.5-84.1). Twenty-five patients were male gender (42%) and 27 were female (45%). For eight patients the gender remained inconclusive. Data about histology, TNM stages and differentiation of the tumors are summarized in Supplementary Table 1. Due to the complete anonymization of all patients after merging with clinical data, lacking data could not be added. An overview of the investigated targets and pathways is summarized in Figure 1. Figure 1A delineates the investigated DNA-repair members and Figure 1B the folic acid pathway.

### Folic acid metabolism

**Figure 2:**
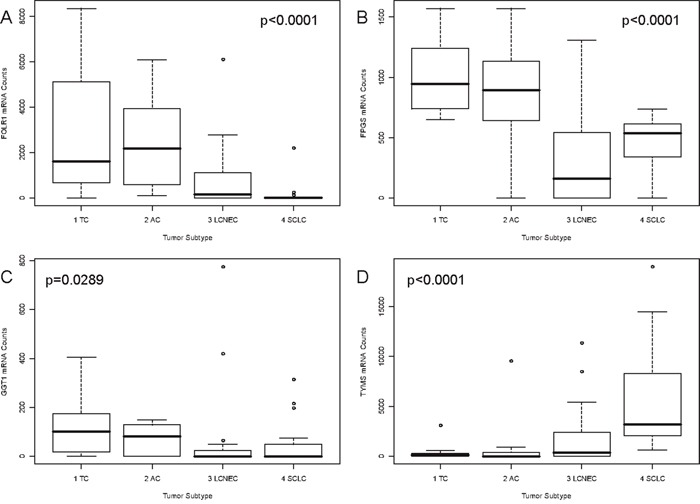
Correlation of *FOLR1*, *FPGS*, *GGT1* and *TYMS* mRNA expression and tumor subtype Associations between the tumor subtype and gene expression of **A.** *FOLR1*, **B.** *FPG*S, (upper line), **C.** *GGT1* and **D.** *TYMS* (lower line) are pictured as boxplots. On the x-axis the four investigated tumor subtypes are depicted. The y-axis shows the mRNA counts measured by the nCounter technology. The p-value is based on a Kruskal-Wallis rank sum test and is rounded to the fourth decimal place. *FOLR1* and *GGT1* expression decreased with increasing malignancy from TC to SCLC. The opposite was found for *TYMS*. *FPGS* showed higher expression in carcinoids than in carcinomas.

With respect to folic acid metabolism, carcinoids showed significantly higher expression than carcinomas for *FOLR1* (p<0.0001), *FPGS* (p<0.0001) and *GGT1* (p=0.0289). The opposite was found for *TYMS* (p<0.0001). These results are summarized in Figure 2A-2D. In line with that, *FOLR1* (p<0.0001), *FPGS* (p<0.0001), *GGT1* (p=0.0366) and *TYMS* (p<0.0001) associated with grade of differentiation as shown in Supplementary Figure S1A-S1D. Furthermore, decreasing *FOLR1* and *FPGS* expression associated significantly with increasing malignancy of the investigated NELC with respect to TNM classification (TNM stage: *FOLR1* p=0.0049, spread to regional lymph nodes: *FOLR1* p=0.0001, *FPGS* p=0.0038). The results are depicted in Supplementary Figure S2A-S2C. Both showed significantly higher expression in female patients (*FOLR1* p=0.0037, *FPGS* p=0.0489) (data not shown).

Besides, further significant correlations for members of the folic acid pathway were found for TNM criteria and are summarized in Table 1.

**Table 1:** Summary of the statistical tests performed and obtained significances

Statistical Test		
**Tumor Type**	**Kruskal-Wallis rank sum test**	**Chi-square**
*FOLR1*	p<0.0001	27.16
*FPGS*	p<0.0001	29.24
*GGT1*	p=0.0289	9.03
*TYMS*	p<0.0001	23.69
*FOLR1/TYMS* Ratio	p<0.0001	36.44
*FOLR1/SLC19A1* Ratio	p<0.0001	24.94
*FPGS/TYMS* Ratio	p<0.0001	32.77
*FPGS/GGT1 Ratio*	p=0.1	
*ERCC1*	p=0.0021	14.69
*MLH1*	p<0.0001	16.61
*XRCC1*	p=0.0131	10.75
*MSH6*	p=0.0064	12.29
*MLH1/MSH2* Ratio	p<0.0010	16.34
		
	**Pearson's Chi-squared test**	**X-squared**
*FOLR1/TYMS* Phenotype	p<0.0001	47.34
*FGPS/TYMS* Phenotype	p<0.0001	47.32
*MLH1/MSH2* Phenotype	p=0.0014	27.10
		
**Grade of Differentiation**	**Spearman's rank correlation rho**	**rho**
*FOLR1*	p<0.0001	−0.6421
*FPGS*	p<0.0001	−0.6501
*TYMS*	p<0.0001	0.6329
*GGT1*	p=0.0366	−0.2825
*ERCC1*	p<0.007	−0.3599
*MLH1*	p=0.0046	−0.3766
*MSH6*	p=0.0210	0.3105
		
**Tumor Stage**	**Spearman's rank correlation rho**	**rho**
*FOLR1*	p=0.0049	−0.4076
*ERCC1*	p=0.0147	−0.3575
*MLH1*	p=0.0214	−0.3383
*XRCC1*	p=0.0226	−0.3357
**Spread to Lymph Nodes (N-Stage)**	**Spearman's rank correlation rho**	**rho**
*FOLR1*	p=0.0001	−0.5192
*FPGS*	p=0.0038	−0.4021
*MLH1*	p=0.0111	−0.3562
		
**Gender**	**Exact Wilcoxon Mann-Whitney Rank Sum Test**	**Z-value**
*FOLR1*	p=0.0037	2.87
*FPGS*	p=0.0489	1.97
*ERCC1*	p=0.0408	2.04
*MLH1*	p=0.0308	2.15
*XRCC1*	p=0.0246	2.24
		
**Overall Survival (OS)**	**Likelihood ratio test**	**Hazard Ratio**
Data for 35 patients (10 events)		
*FOLR1*	p=0.0014	1.00
*FPGS*	p<0.0004	1.00
*TYMS*	p=0.0200	0.99
*ERCC1*	p=0.0082	1.00
*MLH1*	p<0.0040	1.00
*XRCC1*	p=0.0263	1.00
*FOLR1/TYMS* Ratio	p<0.0040	1.03
*FOLR1/SLC19A1* Ratio	p=0.0017	1.05
*FPGS/TYMS* Ratio	p<0.0001	2.54
		
**Progression-Free Survival (PFS)**	**Score (logrank) test**	**Hazard Ratio**
Data for 11 patients (8 events)		
*FOLR1*	p=0.0049	0.99
*ERCC1*	p=0.0026	0.99
*FOLR1/SLC19A1* Ratio	p=0.0223	0.69
*FOLR1/TYMS* Ratio	p=0.0200	0.26
*FPGS/TYMS* Ratio	p=0.0225	<0.01

**Figure 3:**
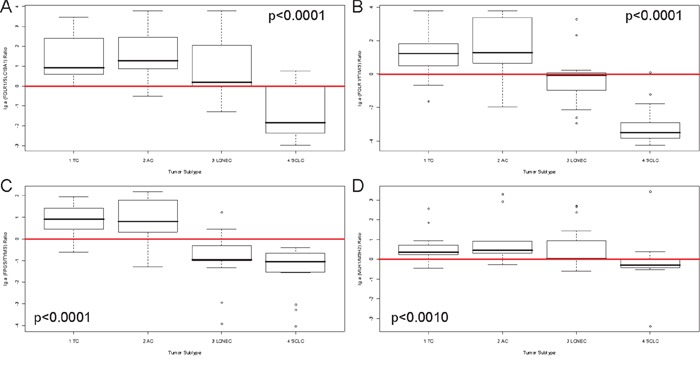
Ratios of members of folic acid metabolism and DNA repair discriminate different subtypes of pulmonary neuroendocrine tumors The natural logarithmical scaled ratios for **A.** *FOLR1/SLC19A1*, **B.** *FOLR1/TYMS*, **C.** *FPGS/TYMS* and **D.** *MLH1/MSH2* are shown (y-axis) in correlation to the four investigated tumor subtypes (x-axis). The p-value is based on a Kruskal-Wallis rank sum test and is rounded to the fourth decimal place. A red line depicts the x-axis at the zero-point. The ratio depicted in **A.** differentiate SCLC from the other entities, indicating that SCLC use *SLC19A1* as predominant receptor for uptake of folic acids. The ratios in **B.** and **C.** identify higher *FOLR1* and *FPGS*, but lower *TYMS* expression in carcinoids. In carcinomas, higher *TYMS* expression is found, whereas *FOLR1* and *FPGS* are considerably reduced.

After identification of *FOLR1* and *FPGS* as markers for more aggressive NELC, further correlations between folic acid pathway members were investigated. A ratio between *FOLR1* and *SLC19A1* was calculated to identify the prominent uptake mechanism for each tumor subtype. The ratio correlated significantly with tumor type (p<0.0001, Figure 3A) and identified *FOLR1* to mediate the uptake in TC to LCNEC with an inverted ratio in SCLC, where *SLC19A1* seems to mediate the folic acid uptake. Next, the correlation between uptake (*FOLR1*), activation (*FPGS*) and utilization (*TYMS*) was investigated by calculating a ratio between *FOLR1* and *TYMS* as well as *FPGS* and *TYMS*. These ratios were calculated to determine the cellular throughput of the folic acid metabolism and associated significantly with tumor type (both p<0.0001, Figure 3B-3C). A misbalance was seen between carcinoids and carcinomas. In contrast to carcinoids, carcinomas seem to have faster utilization via *TYMS* than their uptake can provide. The ratio between activation of folic acid via *FPGS* and inactivation via *GGT1* was also tested, but reached no statistical significance (data not shown).

**Figure 4:**
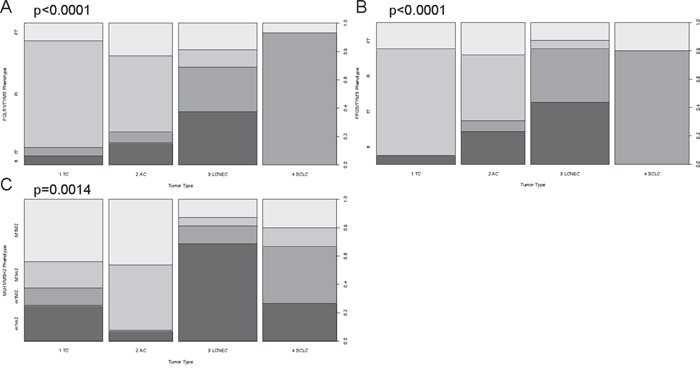
Phenotypic sorting reveals differential expression patterns between carcinoids and carcinomas The phenotypic distribution for **A.** *FOLR1/TYMS*, **B.** *FPGS/TYMS* and **C.** *MLH1/MSH2* is shown for each tumor entity. Phenotypic sorting was performed by classifying expression below the median with a lowercase letter (f, t, m1, m2) and expression above the median with a capital letter (F, T, M1, M2): (*FOLR1* (median=313 counts), *FPGS* (median=665 counts), *TYMS* (median=341 counts), *MLH1* (median=850 counts), *MSH2* (median=612 counts)). **A.** and **B.** show the folic acid phenotypes, whereas **C.** depicts the DNA repair phenotype. On the x-axis the four investigated tumor types are displayed. The y-axis depicts the percentaged distribution of each phenotype. The p-value is based on a Pearson's Chi-squared test and is rounded to the fourth decimal place. Approximately 90% of all TC and more than 75% of AC belong to the Ft- or FT-phenotype indicating high expression of *FOLR1* and *FPGS*, but low *TYMS* predestining them for a pemetrexed treatment. LCNEC present in equal shares ft- and fT-phenotypes making them resistant against pemetrexed. In >95% of SCLC the fT-phenotype was present revealing why pemetrexed therapy is no option for this entity. With respect to DNA repair, LCNEC and SCLC present with the m1m2- and m1M2-phenotype in >65% of all cases, which is linked to platin resistance.

Furthermore, a phenotypic pathway sorting was performed. The rationale to test for certain phenotypes was their ability to differentiate the distinct tumor types by system biological means. The samples were classified as either having mRNA expression counts above the median (depicted by capital letters e.g. F or T) or below median (depicted by lowercase letters e.g. f or t). Figure 4A-4B depicts the folic acid phenotypes for A) *FOLR1* (median=313 counts) and *TYMS* (median=341 counts) and B) *FPGS* (median=665 counts) and *TYMS*. With respect to these phenotypes, SCLC showed one predominant pattern (fT), indicating lower folic acid uptake and activation compared to their turnover rate. In contrast, AC and TC predominantly associated with the Ft-phenotype having high uptake and activation but a considerably low utilization of folic acid. LCNEC were more heterogenic, but two-third of all LCNEC cases associated with ft- or fT-phenotype indicating a low uptake and activation rate.

**Figure 5:**
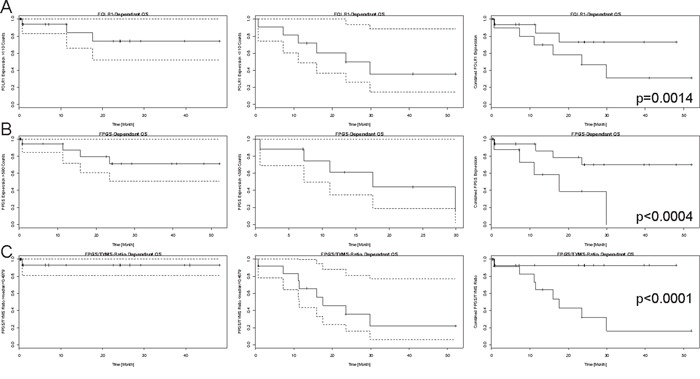
Kaplan-Meier curves for mRNA-expression dependant overall survival Each line presents the single-fractional and combined Kaplan-Meier curves for **A.** *FOLR1*, **B.** *FPGS* and **C.** *FPGS/TYMS* ratio. The single-fractional curves on the left side display the expression-dependant survival above the chosen threshold (*FOLR1*=110 mRNA counts, *FPGS*=590 mRNA counts, and *FPGS/TYMS* ratio=median=0.4979, empirically determined), whereas the single-fractional curves in the middle depict the survival curve for expression above the mentioned expression. Both curves contain the 95% confidence interval, which is pictured by the dashed lines. The combined survival curve is shown on the right side and contains the p-value, which was obtained by using the COXPH-model and is rounded to the fourth decimal place. The x-axis is a time line and the y-axis displays the number of events in a percentaged scale. Higher expression of *FOLR1* and *FPGS* correlated with prolonged survival and a higher *FPGS* expression than *TYMS* expression was identified as further positive prognostic marker.

*FOLR1* (p=0.0014, HR=1) and *FPGS* (p<0.0004, HR=1) associated significantly with OS, but due to a hazard ratio of one they have negligible clinical relevance (shown in Figure 5A-5B). Additionally, the ratios of *FOLR1* and *SLC19A1* (p=0.0017, HR=1.05), *FOLR1* and *TYMS* (p<0.0040, HR=1) as well as *FPGS* and *TYMS* (p<0.0001, HR=2.54, Figure 5C) associated with overall survival (further data not shown due to repetitiveness to Figure 5A-5C).

For PFS, *FOLR1* (p=0.0049, HR=1), *FOLR1* and *TYMS* ratio (p=0.0200, HR=0.26), *FOLR1* and *SLC19A1* ratio (p=0.0223, HR=0.69) as well as *FPGS* and *TYMS* ratio (p=0.0225, HR<0.01) reached statistical significance (data not shown due to repetitiveness to Figure 5A-5C).

Assessment of the supposed ratios showed a high merit with clinically relevant hazard ratios compared to single marker evaluation. Especially, the *FPGS/TYMS* ratio presented with a high discriminative power between poor and good prognosis and response to therapy.

The supposed phenotypes were not correlated with survival data due to the low number of cases per phenotype.

### DNA-repair members

Members of the DNA-repair pathway correlated significantly with tumor type (*ERCC1* p=0.0021, *MLH1* p<0.0001, *XRCC1* p=0.0131, *MSH6* p=0.0064) (data not shown) as well as an *MLH1/MSH2* ratio (p<0.0010), which was calculated as stated above (depicted in Figure 3D). Furthermore, the *MLH1*/*MSH2* phenotype was assessed as mentioned above (*MLH1*: median=850 counts; *MSH2*: median=612 counts) and reached significance with respect to tumor type (p=0.0014) as shown in Figure 4C. The M1m2- and M1M2-phenotype correlated with carcinoids (60% of TC and >90% of AC), whereas carcinomas showed mostly reduced MSH1 expression (m1m2 or m1M2 in >80% of LCNEC and >65% of SCLC). *ERCC1* (OS: p=0.0082, HR=1, PFS: p=0.0026, HR=1, data not shown) associated with survival. These and additional results are summarized in Table 1.

## DISCUSSION

The mRNA level of eight genes involved in the folic acid metabolism and five genes of DNA damage repair were investigated in 60 NELC to identify subgroups that could benefit from cisplatin/pemetrexed therapy or show mechanisms of resistance against these regimens. One reason for the lack of comprehensive studies in NELC is the rather low frequency of TC and AC in the general lung cancer patient population. Due to the high number of lung cancer cases diagnosed at our tertiary cancer centre, a sufficient number of pulmonary TC and AC cases were available for our study.

Folate receptor 1 (the protein of the *FOLR1* gene) is usually absent in SCLC and infrequently expressed in LCNEC [44, 45], in accordance with our results. Overall, low *FOLR1* expression was identified as marker for more aggressive NELC and associated with shorter OS and PFS. Although survival data for the investigated collective was limited to 35 patients for overall (OS) and 11 patients for progression-free survival (PFS) (10 and 8 events per group, respectively), we considered the results to be of interest due to their consistency to the above mentioned results.

Our group has recently reported the predictive value of FOLR1 expression in NSCLC patients receiving pemetrexed-based chemotherapy [46, 47], indicating *FOLR1* as potent biomarker in lung cancer.

We observed a significantly different *TYMS* gene expression between AC, TC, LCNEC, and SCLC with significantly higher expression especially in SCLC and particularly LCNEC. Similar results were found in retrospective studies using quantitative real-time PCR and immunohistochemistry [13, 48]. Pemetrexed is a multitargeted antifolate that acts against TYMS, DHFR, GART and ATIC and is approved for the treatment of metastatic and unresectable non-squamous NSCLC [49]. Elevated expression of TYMS is believed to be one reason for the low efficacy of pemetrexed in SCLC [50]; similarly, squamous cell carcinomas of the lung express higher amounts of *TYMS* [51], which show poor response rates to pemetrexed-based therapies [49, 52]. Putatively, low levels of FPGS might be predictive for the efficacy of pemetrexed [21, 49].

In addition to the single marker assessment, which already revealed *FOLR1* and *FPGS* as marker for aggressiveness and survival, the phenotypic sorting gave additional information and allowed for more detailed risk stratification of the investigated NELC. Phenotyping with respect to the folic acid metabolism revealed that SCLC show resistance to a pemetrexed therapy, because of the fT phenotype (low *FOLR1* and *FPGS*, high *TYMS*). A large randomized phase III clinical trial (GALES; Global Analysis of pemetrexed in SCLC Extensive Stage) had to be aborted due to futility of pemetrexed plus carboplatin regimen compared to the standard etoposide plus carboplatin approach [53, 54], which can be explained by the supposed folic acid metabolism phenotype.

In contrast, carcinoids present predominantly with the Ft-phenotype indicating high *FOLR1* and *FPGS*, but low *TYMS* gene expression levels. Especially, FOLR1 overexpression seems to be a tumor marker in a tissue-dependant manner and has gained interest for targeted therapies via antibodies (e.g. farletuzumab) or new folic acid-drug conjugates (e.g. vintafolide) for selective inhibition, which were tested in clinical trails [55]. Hence, the Ft-phenotype found in TC/AC provides the rationale for the administration of pemetrexed for the treatment of unresectable or metastatic TC and AC, which commonly occur at a very low frequency (1 TC and 2 AC in our study collective). Discussions about the efficacy of cisplatin/etoposide in this setting are ongoing [56] and no standard-of-care for chemotherapy exists. Three of the investigated carcinoids showed spread to regional lymph nodes indicating an aggressive subtype of a rather low-grade tumor. The phenotypic sorting with respect to folic acid metabolism revealed that two of them showed *FOLR1* expression above the median expression (Ft-phenotype) and also the third one showed a misbalance towards *FOLR1* (FT-phenotype, with only <3% above the median expression level of *TYMS*) indicating a potential benefit of an anti-folate therapy e.g. by pemetrexed or a new targeted therapy concept (vintafolide, farletuzumab) [55]. Of note, AC, TC and LCNEC belong to NSCLC [5], although of their neuroendocrine differentiation, and pemetrexed is approved for non-squamous NSCLC [49], making pemetrexed a reasonable therapy option.

With respect to the DNA-repair members, the lowest *MLH1* expression was found in LCNEC. A comparison of *MLH1* expression between pulmonary adenocarcinomas, squamous cell carcinomas and LCNEC was reported showing normal *MLH1* expression (compared to normal bronchial mucosa) in 13 out of 18 LCNEC specimens and reduced expression in the remaining 5 specimens [57]. Reduction of MLH1 expression was reported to be a driver of platin resistance [58, 59] indicating that high-grade NELC are more prone to platin resistance than carcinoids, according to our results. Elevated expression of MSH2 was identified to contribute to such resistance mechanism [59]. The phenotypic sorting with respect to DNA repair revealed the negative prognostic m1m2- and m1M2-phenotype to be associated with high-grade tumors indicating overall reduced *MLH1* expression in high-grade NELC.

The NanoString nCounter system is a reproducible, sensitive and specific method that can detect even low abundance mRNAs that are below the detection limit of microarrays [35]. Additionally, the method is able to allow analysis of FFPE tissue yielding similar results as using fresh-frozen tissue for RNA investigations [60]. Due to the advanced stage at diagnosis, usually biopsies were collected from LCNEC and SCLC patients yielding rather small specimens and a huge part of these specimens was used for RNA extraction. We decided not to perform RT-PCR, because the NanoString nCounter system has already been validated in several publications [35, 36, 39, 41, 60]. A weakness of our current work is the lack of immunohistochemical investigations, which use antibodies specifically directed against functionally active enzymes. Discussions about their specificity and particularly about their ability to detect functionally active enzymes are ongoing [61]. Besides, validation of the nCounter technology by other techniques (e.g. qPCR, IHC, blotting etc.) may not be important as NanoString has launched an FDA- and CE-IVD-approved mRNA expression test (Prosigna) for the investigation of the PAM50-gene signature in FFPE-derived samples from breast cancer patients [62].

To summarize, gene expression of enzymes involved in folic acid metabolism as well as repair of DNA damage showed strong associations with the tumor entity in neuroendocrine tumors of the lung. Associations between the differentiation of these tumors, lymph node invasion and prognostic or predictive biomarkers could be identified. The supposed multi-marker assessment (phenotypic sorting) and system biological analysis can enhance risk stratification and provide additional guidance for therapy identification, also beyond lung cancer of the neuroendocrine subtype.

## MATERIALS AND METHODS

### Study design

For this biomarker exploratory study, sixty different formalin-fixed paraffin-embedded (FFPE) tumor specimens representative for each tumor entity (16 TC, 13 AC, 16 LCNEC, and 15 SCLC) were used for mRNA expression analysis. The initial diagnosis was made according to the *WHO Classification Of Tumors* [5], based on immunohistochemistry and confirmed by two experienced pathologists (JWO, THA). Only tumor specimens with a minimum of infiltration by lymphocytes or stromal cells and which were received from patients before any chemo- or radiotherapy were considered. The study included only patients with pulmonary neuroendocrine tumors, who were treated at the West German Cancer Centre between 2005 and 2011. Clinicopathological data included age, gender, histology, stages (Supplementary Table 1). Tumor staging was based on the tumor, node, and metastasis (TNM) staging system (7^th^ edition) as proposed by the International Association for the Study of Lung Cancer (IASLC) [40]. Progression-free survival (PFS) was calculated from the date of diagnosis until progression or loss to follow-up during any treatment. Overall survival (OS) was defined as the time between diagnosis until the date of death, or the date of last follow-up. Patients were censored at the last follow-up if still alive or lost to follow-up. Surveillance of PFS/OS for this study was stopped on August 31, 2014. The retrospective study was approved by the Ethics Committee of the Medical Faculty of the University Duisburg-Essen (identifier: 13-5382-BO). The investigation conforms to the principles outlined in the declaration of Helsinki.

### RNA extraction and RNA integrity assessment

According to the manufacturer's recommendations, three to five FFPE sections with a thickness of 4 μm per slide were deparaffinised with xylene prior to RNA extraction using the RNeasy FFPE kit (Qiagen, Venlo, Netherlands). RNA concentration was measured using a Nanodrop 1000 instrument (Thermo Fisher Scientific, WA, USA). RNA integrity was assessed using an Agilent 2100 Bioanalyzer (Agilent Technologies, CA, USA) at the NanoString nCounter Core Facility at the University of Heidelberg (Heidelberg, Germany). Smear analysis was performed using the Agilent 2100 expert software to determine the proportion of RNA ≥300 nt within a given sample.

### NanoString CodeSet design and expression quantification

Important genes of the folic acid metabolism (eight genes: *ATIC*, *DHFR*, *FOLR1*, *FPGS*, *GART*, *GGT1*, *SLC19A1*, *TYMS*) and DNA damage repair (five genes: *ERCC1*, *MLH1*, *MSH2*, *MSH6*, *XRCC1*) were included in the CodeSet. The complete CodeSet was designed to contain a total of 91 genes with different signature genes for each tumor entity and some results have been published previously [36, 39, 41]. Three potential reference genes (*ACTB, GAPDH, and HPRT1*) were also included in the CodeSet for biological normalization purposes [38, 42]. Probe sets for each gene in the CodeSet were designed and synthesized at NanoString Technologies (Seattle, WA, USA). Total RNA (100 ng) including mRNA and miRNA was measured at the NanoString nCounter Core Facility at the University of Heidelberg, Germany.

### NanoString data processing and statistical analysis

Raw NanoString counts for each gene were subjected to a technical normalization taking positive and negative probes into account. A background correction was carried out by subtracting average negative-control counts plus two-times standard deviation from each target counts. After this procedure a biological normalization using reference genes was performed by choosing appropriate reference genes based on the geNorm algorithm [43]. All statistical analyses were calculated with the R statistical programming environment (v3.1.1.). For dichotomous factors such as gender and expression level the Wilcoxon Mann-Whitney rank sum test was applied. For variables with more than two categories the Kruskal-Wallis test was done. Associations between gene expression of tested genes and associations between gene expression and TNM-criteria were analyzed by Spearman's rank correlation test. Two-dimensional contingency tables were analyzed using the Pearson's Chi-squared test.

Kaplan-Meier analysis was done for the assessment of associations between gene expression and survival data. Significant survival differences between groups were verified by COXPH-model using a confidence interval of 95% for the Wald-test, likelihood-ratio test and Score (logrank) test. The level of statistical significance was defined as p≤0.05.

## SUPPLEMENTARY FIGURES AND TABLES





## Abbreviations

AC: atypical carcinoids
ALK: anaplastic lymphoma receptor tyrosine kinase
CI: confidence interval
DHFR: dihydrofolate reductase
EGFR: epidermal growth factor receptor
ERCC1: excision repair cross-complementing group 1
FOLR1: folate receptor 1
FFPE: formalin-fixed, paraffin-embedded
FPGS: folylpolyglutamate synthase
GART: phosphoribosylglycinamide formyltransferase, phosphoribosylglycinamide synthetase, phosphoribosylaminoimidazole synthetase or glycinamide ribonucleotide formyltransferases
GGT1: gamma-glutamyltransferase 1
HR: hazard ratio
LCNEC: large-cell neuroendocrine cancer
MLH1: mutL homolog 1
MMR: mismatch repair
MSH2: mutS homolog 2
nt: nucleotides
NELC: neuroendocrine lung cancer
NER: nucleotide excision repair
NSCLC: non-small cell lung cancer
OS: overall survival
PFS: progression-free survival
SLC19A1: solute carrier family 19 (folate transporter), member 1
SLC46A1: solute carrier family 46 (folate transporter), member 1
SCLC: small cell lung cancer
TYMS: thymidylate synthetase
TC: typical carcinoids
WHO: world health organization
XRCC1: x-ray repair complementing defective repair in Chinese hamster cells 1.
