# Supplementary material for: Folic-acid metabolism and DNA-repair phenotypes differ between neuroendocrine lung tumors and associate with aggressive subtypes, therapy resistance and outcome
Source: Oncotarget. 2016 Feb 26;7(15):20166–79. doi: 10.18632/oncotarget.7737 (PMC4991445; doi:10.18632/oncotarget.7737)
Supplement: Supplementary file 1 [file oncotarget-07-20166-s001.pdf]

SUPPLEMENTARY FIGURES AND TABLE

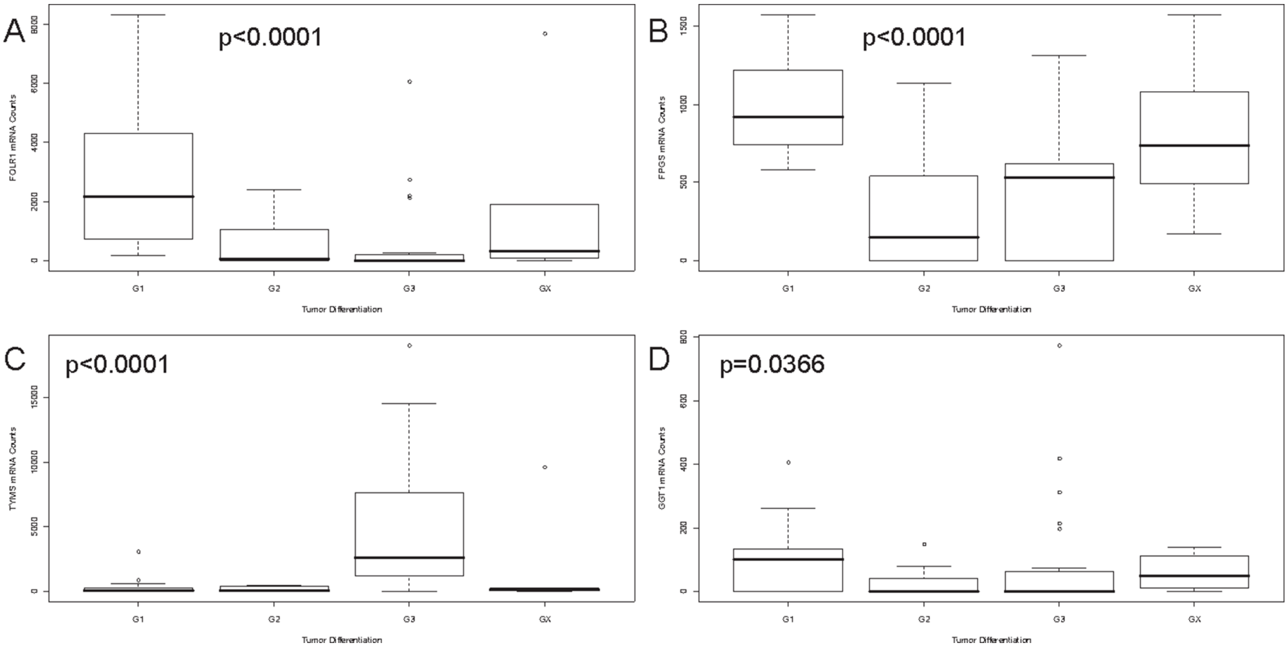

Supplementary Figure S1: Correlation of *FOLR1*, *FPGS*, *GGT1* and *TYMS* mRNA expression and grade of differentiation. Associations between grade of differentiation and gene expression of **A.** *FOLR1*, **B.** *FPGS*, (upper line), **C.** *TYMS* and **D.** *GGT1* (lower line) are pictured as boxplots. On the x-axis grade of differentiation is plotted. The y-axis shows the mRNA counts measured by the nCounter technology. The p-value is based on a Kruskal-Wallis rank sum test and is rounded to the fourth decimal place. *FOLR1*, *FPGS* and *GGT1* showed highest expression in low-grade tumors. *TYMS* expression was predominantly found in G3-tumors.

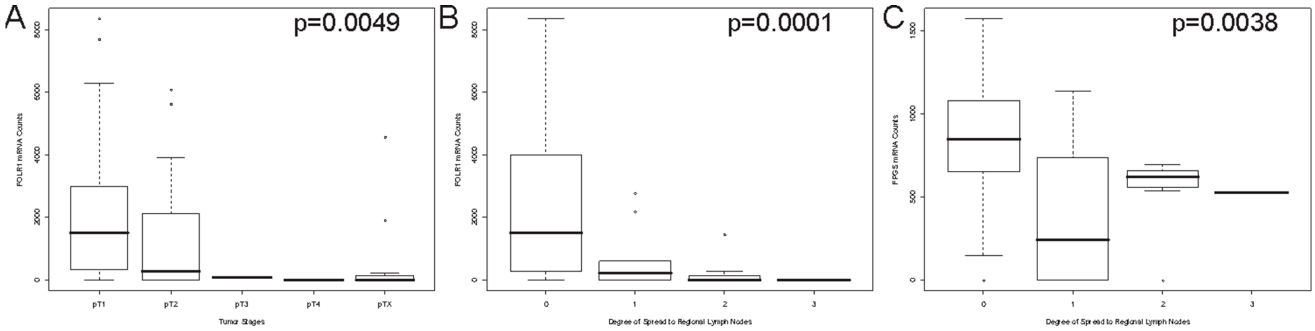

Supplementary Figure S2: Correlation of *FOLR1* and *FPGS* mRNA expression and lymph node invasion and *FOLR1* expression and tumor stage. Associations between gene expression of *FOLR1* and **A.** tumor stage and **B.** lymph node invasion as well as *FPGS* expression and **C.** lymph node invasion are pictured as boxplots. On the x-axis the T-stages or N-stages are depicted. T-stage ptX indicates unknown stage. The y-axis shows the mRNA counts measured by the nCounter technology. The p-value is based on a Kruskal-Wallis rank sum test and is rounded to the fourth decimal place. *FOLR1* expression decreased dramatically with more aggressive tumors with respect to tumor stage and degree of spread to regional lymph nodes. *FPGS* showed highest expression in tumors without lymph node invasion.

Supplementary Table 1: Clinicopathological data of lung cancer patients. TNM stages refer to the initial diagnosis of lung cancer. Note that percentages may not total 100 due to rounding off**.**

See Supplementary File S1
