# Supplementary material for: Folic-acid metabolism and DNA-repair phenotypes differ between neuroendocrine lung tumors and associate with aggressive subtypes, therapy resistance and outcome
Source: Oncotarget. 2016 Feb 26;7(15):20166–79. doi: 10.18632/oncotarget.7737 (PMC4991445; doi:10.18632/oncotarget.7737)
Supplement: Supplementary file 2 [file oncotarget-07-20166-s002.doc]

Supplementary Table 1

| **Epidemiologic and clinicopathological data** | **Number of patients (%)** |
| --- | --- |
|  | 60 (100%) |
|  |  |
| **Age** |  |
| Median age at initial diagnosis in years (range) | 59 (20-84) |
|  |  |
| **Gender** |  |
| Female | 27 (45 %) |
| Male | 25 (42 %) |
| Unknown | 8 (13 %) |
|  |  |
| **T-stage (at initial diagnosis)** |  |
| T1 | 29 (48%) |
| T2 | 14 (23%) |
| T3 | 1 (2 %) |
| T4 | 2 (3 %) |
| Tx | 14 (23 %) |
|  |  |
| **N-stage (at initial diagnosis)** |  |
| N0 | 33 (55 %) |
| N1 (1 AC, 6 LCNEC, 2 SCLC) | 9 (15 %) |
| N2 (1 TC, 1 AC, 1 LCNEC, 4 SCLC) | 7 (12 %) |
| N3 (1 SCLC) | 1 (2 %) |
| Nx | 10 (17 %) |
|  |  |
| **M-stage (at initial diagnosis)** |  |
| M0 | 48 (80 %) |
| M1 | 2 (3 %) |
| Mx | 10 (17 %) |
|  |  |
| **Histologic subtype** |  |
| Typical carcinoid | 16 (27 %) |
| Atypical carcinoid | 13 (22 %) |
| Large cell neuroendocrine cancer | 16 (27 %) |
| Small cell lung cancer | 15 (25 %) |
|  |  |
| **Histological grading** |  |
| G1 | 23 (38%) |
| G2 | 7 (12 %) |
| G3 | 16 (27 %) |
| Gx | 14 (23 %) |
